# A *Lactococcus lactis* expression vector set with multiple affinity tags to facilitate isolation and direct labeling of heterologous secreted proteins

**DOI:** 10.1007/s00253-017-8524-x

**Published:** 2017-10-02

**Authors:** Francisco Romero Pastrana, Jolanda Neef, Jan Maarten van Dijl, Girbe Buist

**Affiliations:** Department of Medical Microbiology, University of Groningen, University Medical Center Groningen, Hanzeplein 1, P.O. Box 30001, 9700 RB Groningen, The Netherlands

**Keywords:** *Lactococcus lactis*, Expression vector, Strep-tag, AVI-tag, *Staphylococcus aureus*

## Abstract

The gram-positive bacterium *Lactococcus lactis* is a useful host for extracellular protein production. A main advantage of *L. lactis* over other bacterial expression systems is that lactococcal cells display low levels of autolysis and proteolysis. Previously, we developed a set of vectors for nisin-inducible extracellular production of N- or C-terminally hexa-histidine (His_6_)-tagged proteins. The present study was aimed at expanding our portfolio of *L. lactis* expression vectors for protein purification and site-specific labeling. Specifically, we present two new groups of vectors allowing N- or C-terminal provision of proteins with a Strep-tag II or AVI-tag. Vectors for AVI-tagging encode an additional His_6_-tag for protein purification. Another set of vectors allows removal of N-terminal Strep- or His_6_-tags from expressed proteins with the tobacco etch virus protease. Two possible applications of the developed vectors are presented. First, we show that Strep-tagged LytM of *Staphylococcus aureus* in the growth medium of *L. lactis* can be directly bound to microtiter plates coated with an affinity reagent and used for enzyme-linked immunosorbent assays. Second, we show that the AVI-tagged Sle1 protein from *S. aureus* produced in *L. lactis* can be directly biotinylated and fluorescently labeled. The fluorescently labeled Sle1 was successfully applied for *S. aureus* re-binding studies, allowing subcellular localization by fluorescence microscopy. In conclusion, we have developed a set of expression vectors that enhances the versatility of *L. lactis* as a system for production of proteins with tags that can be used for affinity purification and site-specific protein labeling.

## Introduction

The gram-positive bacterium *Lactococcus lactis* is known to be a suitable host for the expression and secretion of heterologous proteins (Pontes et al. 2011). In most *L. lactis* expression systems, the production of proteins is induced using the nisin-inducible (NICE) system. Here, the expression of a target gene is directed by the *nisA* promoter, which is activated in the presence of the food-grade lantibiotic nisin that activates the NisRK two-component regulatory system (Ruyter et al. 1996; Kuipers et al. 1998). Different vectors using the NICE system have been constructed for both cytoplasmic and secreted production of (heterologous) proteins (Mierau and Kleerebezem 2005). For extracellular production, proteins were secreted via the Sec secretion machinery using the signal peptide of the lactococcal protein Usp45 (Borrero et al. 2011; Ng and Sarkar 2013). Recently, a set of vectors suitable for inducible extracellular protein production of N- or C-terminally hexa-histidine (His_6_)-tagged proteins was published (Neef et al. 2015).

The His_6_-tag is one of the most widely used tags as it allows efficient one-step purification of tagged proteins by metal affinity chromatography (Jones et al. 1995). However, this tag can have several drawbacks. For example, there may be many contaminating proteins (Lichty et al. 2005) and the His_6_-tag may lead to protein dimerization (Wu and Filutowicz 1999), instability, or degradation of tagged proteins (Rosales and Lee 2000). Also, His_6_-tags may interfere with ligand or substrate binding (Fonda et al. 2002). Therefore, the use of alternative protein tags could increase the chances of obtaining efficient protein production and purification and, at the same time, provide opportunities for direct labeling applications. For the isolation or labeling of expressed proteins, several tags have been used in *L. lactis*, such as the Strep-tag (Lubelski et al. 2006; Frelet-Barrand et al. 2010; Bernaudat et al. 2011), Flag-tag (Diep et al. 2007), Myc-tag (Bosma et al. 2006; Dieye et al. 2010; Visweswaran et al. 2013), and AVI-tag (Seeger et al. 2012; Neef et al. 2014). Only for the last two tags, secretion of heterologous tagged proteins was demonstrated in *L. lactis*.

The Strep-tag II system is based on an 8-amino-acid peptide tag (WSHPQFEK) with reversible high affinity to Strep-Tactin (an engineered form of streptavidin). It is derived from the well-known extremely high-affinity binding of biotin to streptavidin (Skerra and Schmidt 2000). The short, biologically inert, and proteolytically stable peptide tag allows purification of biologically active Strep-tagged fusion proteins under mild conditions (Schmidt and Skerra 2007). Cytoplasmic expression of a C-terminal Strep-tag fusion in *L. lactis* using the NICE system was shown for the LmrR protein (Lubelski et al. 2006).

The AVI-tag system involves a 15-amino-acid peptide (GLNDIFEAQKIEWHE) recognized by the biotin ligase BirA that catalyzes the amide linkage between biotin and the lysine residue in the AVI-tag peptide (Cull and Schatz 2000). Production in *L. lactis* of secreted staphylococcal proteins with an N-terminal AVI-tag for site-specific labeling with biotin has been reported recently (Neef et al. 2014).

The present study was aimed at expanding our portfolio of *L. lactis* expression vectors. Specifically, we constructed two vector sets by introducing sequences encoding N- or C-terminal AVI- or Strep-tags. The functionality of these vectors was demonstrated by expressing and secreting the tagged staphylococcal reporter proteins LytM and Sle1. The produced and exported proteins were used for rapid immune screening, and direct labeling for detection of localized binding on staphylococcal cells, respectively.

## Materials and methods

### Bacterial strains and growth conditions

Strains and plasmids are listed in Table 1. *L. lactis* strains were grown at 30 °C in M17 broth (Oxoid Limited, Hampshire, UK) supplemented with 0.5 or 2% glucose (*w*/*v*) (GM17). Standing cultures were supplemented with 0.5% glucose. For optimal production of reporter proteins, the *L. lactis* cells were grown in medium supplemented with 2% glucose with shaking (250 rpm). When necessary, the medium was supplemented with chloramphenicol (5 μg/ml). The *Staphylococcus aureus* strains USA300 and NCTC8325 were grown overnight at 37 °C, 250 rpm in Tryptone Soy Broth (TSB; Oxoid Limited).

**Table 1:** Bacterial strains and plasmids used in this study

Strain or plasmid	Relevant phenotype(s) or genotype(s)	Reference
Strains		
*L. lactis* PA1001	MG1363 *pepN*::*nisRK*, allows nisin-inducible expression, Δ*acmA* Δ*htrA*	(Bosma et al. 2006)
*S. aureus* USA300	Community-acquired MRSA isolate	ATCC strain BAA-1717 (McDougal et al. 2003)
*S. aureus* NCTC8325	Restriction-deficient derivative of NCTC 8325; cured of all known prophages	(Kreiswirth et al. 1983)
Plasmids		
pNG4110	Cm^R^, containing P_*nisA*_, SS_*usp45*_, N-term His_6_, MCS	(Neef et al. 2015)
pNG4111	Cm^R^, containing P_*nisA*_, SS_*usp45*_, N-term His_6_, TEV site, MCS	(Neef et al. 2015)
pNG4210	Cm^R^, containing P_*nisA*_, SS_*usp45*_, MCS, C-term His_6_	(Neef et al. 2015)
pNG4110S	pNG4110 derivative with N-term Strep-Tag II	This study
pNG4111S	pNG4111 derivative with N-term Strep-Tag II	This study
pNG4210S	pNG4210 derivative with C-term Strep-Tag II	This study
pNG4110A	pNG4110 derivative with C-term AVI-tag, N-term His_6_	This study
pNG4111A	pNG4111 derivative with C-term AVI-tag, N-term His_6_, TEV site	This study
pNG4210A	pNG4210 derivative with N-term AVI-tag, C-term His_6_	This study
pNG4110-*lytM*	Expression of His_6_-LytM	This study
pNG4111-*lytM*	Expression of His_6_-TEV-LytM	This study
pNG4210-*lytM*	Expression of LytM-His_6_	This study
pNG4110S-*lytM*	Expression of Strep-Tag II-LytM	This study
pNG4111S-*lytM*	Expression of Strep-Tag II-TEV-LytM	This study
pNG4210S-*lytM*	Expression of LytM-Strep-Tag II	This study
pNG4110A-*lytM*	Expression of His_6_-LytM-AVI-tag	This study
pNG4111A-*lytM*	Expression of His_6_-TEV-LytM-AVI-tag	This study
pNG4210A-*lytM*	Expression of AVI-tag-LytM-His_6_	This study
pNG4110-*sle1*	Expression of His_6_-Sle1	This study
pNG4111-*sle1*	Expression of His_6_-TEV-Sle1	This study
pNG4210-*sle1*	Expression of Sle1-His_6_	This study
pNG4110S-*sle1*	Expression of Strep-Tag II-Sle1	This study
pNG4111S-*sle1*	Expression of Strep-Tag II-TEV-Sle1	This study
pNG4210S-*sle1*	Expression of Sle1-Strep-Tag II	This study
pNG4110A-*sle1*	Expression of His_6_-Sle1-AVI-tag	This study
pNG4111A-*sle1*	Expression of His_6_-TEV-Sle1-AVI-tag	This study
pNG4210A-*sle1*	Expression of AVI-tag-Sle1-His_6_	This study

*Cm* ^*R*^ chloramphenicol resistance gene, *P*_*nisA*_ nisin-inducible promoter, *His*_*6*_ hexahistidine-tag, *SS*_*usp45*_ signal sequence of *usp45*, *TEV site* cleavage site for tobacco etch virus protease, *MCS* multiple cloning site

### General molecular biology

Enzymes and buffers were obtained from New England Biolabs (NEB, Ipswich, USA). Genomic DNA of *S. aureus* USA300, used as template for all PCR reactions, was isolated with the Genelute bacterial genomic DNA kit (Sigma-Aldrich, Zwijndrecht, The Netherlands) according to the manufacturer’s protocol with minor modifications as described before (Neef et al. 2014). PCR reactions were performed with a Bio-Rad C1000 thermal cycler (Bio-Rad Laboratories, Richmond, CA). Primers used in this study, shown in Table 2, were obtained from Eurogentec (Maastricht, The Netherlands). The *Taq* (Life Technologies, Bleiswijk, The Netherlands) and Phusion Hot Start II (Thermo Fisher Scientific, Wilmington, Delaware, USA) polymerases were used according to the manufacturer’s protocols. PCR products were purified using the High Pure PCR purification kit (Analytic Jena, Jena, Germany). Ligations with T4 DNA ligase and DNA restriction endonuclease digestions were performed following the manufacturer’s protocols (NEB). Plasmids from *L. lactis* cells were extracted using the innuPREP Plasmid Mini Kit (Analytik Jena) with the following modifications: cell pellets were resuspended in solution A with lysozyme (2 mg/ml, Sigma-Aldrich) and incubated for 10 min at 55 °C. Further DNA purification and concentration were performed with the DNA Clean & Concentrator-5 (Zymo Research, Irvine, CA, USA). The Silica Bead DNA Gel Extraction Kit (Thermo Fisher Scientific) was used for DNA isolation from agarose gels. Nucleotide sequence analyses were performed by Eurofins DNA (Ebersberg, Germany). Electrotransformation of *L. lactis* was performed using a Gene pulser (Bio-Rad Laboratories) as described before (Leenhouts and Venema 1993).

**Table 2:** Primers used for the construction of the expression vectors

Primer	5′ → 3′ nucleotide sequence^a^	R.E.
StrepTag.For	CAATGATTTCGTTCGAAGGAACTAC	*Bst*BI
Strep110.Rev	ATATGGATCC TTTCTCGAACTGCGGGTGGCTCCACATGGAGTTTGTGTCAGCGTAAAC	*Bam*HI
Strep111.Rev	ATATGGATCCCTGGAAGTACAGGTTCTCTTTCTCGAACTGCGGGTGGCTCC	*Bam*HI
Strep210.Rev	ATATAAGCTTTTATTTCTCGAACTGCGGGTGGCTCCATGCGGCCGCCTCGAGGAATTCG	*Hin*dIII
StrepPCR.Rev	CTCGAACTGCGGGTGGCTCC	
AVI-tagNotI.fw	*GGCC*GCCATGAGTGGTTTAAACGATATTTTCGAGGCTCAGAAAATCGAATGGCACGAA **TAA**ATCC	
AVI-tagNotI.rev	*GGCC*GGAT**TTA** TTCGTGCCATTCGATTTTCTGAGCCTCGAAAATATCGTTTAAACCACTCATGGC	
AVI-tagBamHI.fw	*GATC*GCCCATGAGTGGTTTAAACGATATTTTCGAGGCTCAGAAAATCGAATGGCACGAAATCATGG	
AVI-tagBamHI.rev	*GATC*CCATGATTTCGTGCCATTCGATTTTCTGAGCCTCGAAAATATCGTTTAAACCACTCATGGGC	
SleI.F1	ATATGGATCCGCTACAACTCACACAGTAAAAC	*Bam*HI
SleI.R1	ATATGCGGCCGCGTGAATATATCTATAATTATTTACTTGGT	*Not*I
SleI.R2	ATATGCGGCCGC **TTA**GTGAATATATCTATAATTATTTACTTGGT	*Not*I
LytM.F1	ATATGGATCCATGGGAGCAGAAACGACAAACACCC	*Bam*HI
LytM.R1	ATATGCGGCCGCTCTACTTTGCAAGTATGACGTTGGG	*Not*I
LytM.R2	ATATGCGGCCGC **TTA**TCTACTTTGCAAGTATGACGTTGGG	*Not*I

^a^Restriction sites are underlined, stop codons are indicated in bold, *Not*I/*Bam*HI-compatible overhangs are indicated in italics, Strep- and AVI-tag encoding sequences are double underlined, the TEV site encoding sequences are dotted underlined

### Construction of expression vectors

**Fig. 1:**
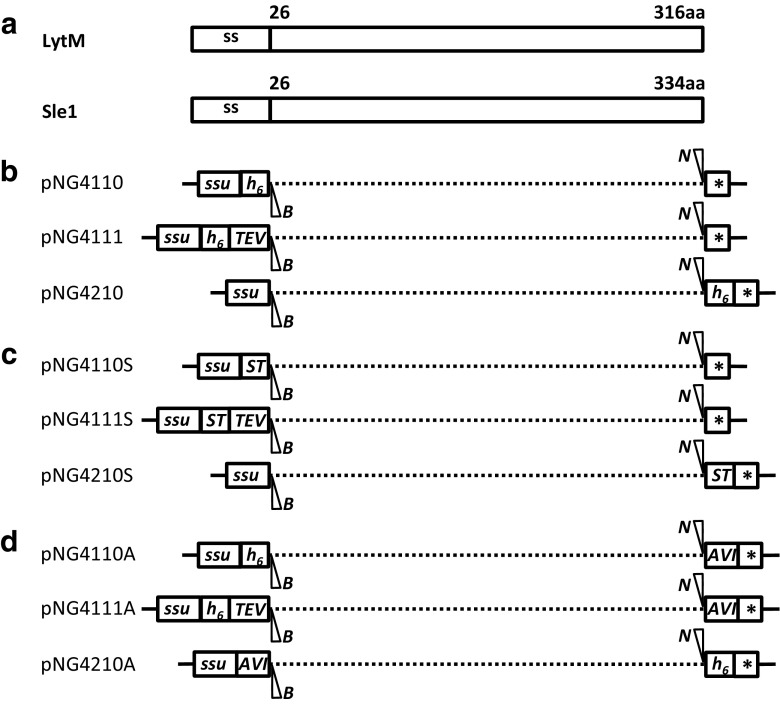
Schematic representation of the expression cassettes present in the different pNG vectors used in this study. **a** Representation of the secretion signal peptides (ss) and mature regions (26–316 and 26–334) of the *S. aureus* proteins LytM and Sle1, respectively. **b** Expression cassettes of the *L. lactis* pNG-vectors, encoding an N-terminal His_6_-tag (pNG4110), a C-terminal His_6_-tag (pNG4210), or a TEV-removable (TEV) N-terminal His_6_-tag (pNG4111). **c** Expression cassettes of the *L. lactis* pNG-vectors, encoding an N-terminal Strep-tag II (pNG4110S), a C-terminal Strep-tag II (pNG4210S), or a TEV-removable (TEV) N-terminal Strep-tag II (pNG4111S). **d** Expression cassettes of the *L. lactis* pNG-vectors, encoding an N-terminal His_6_-tag and a C-terminal AVI-tag (pNG4110A), a C-terminal His_6_-tag and a N-terminal AVI-tag (pNG4210A), or a TEV-removable (TEV) N-terminal His_6_-tag (pNG4111A) and a C-terminal AVI-tag. Positions of the restriction enzyme cleavage sites *Bam*HI (*B*) and *Not*I (*N*), the TEV protease cleavage site (*TEV*), N-terminal or C-terminal His_6_-tag (*h*_*6*_), Strep*-*tag II (*ST*), and AVI-tag (*AVI*) are indicated. ssu signal sequence of the gene for the secreted lactococcal protein Usp45, *stop codon

An overall schematic representation of constructed vectors is shown in Fig. 1. The His_6_-tag present in plasmids pNG4110, pNG4111, and pNG4210 was replaced by the Strep-tag II resulting in the plasmids pNG4110S, pNG4111S, and pNG4210S, respectively. For the construction of plasmid pNG4110S, a PCR fragment was generated using the primers StrepTag.For and Strep110.Rev using plasmid pNG4110 as a template. The PCR product was digested with *Bst*BI and *Bam*HI and ligated to linearized plasmid pNG4110, digested with the same enzymes. The same approach was used to replace the His_6_-tag to obtain plasmids pNG4111S and pNG4210S using specific primers indicated in Table 2, but in this case, the PCR fragments and vector were digested with *Bst*BI and *Hin*dIII. Insertion of the AVI-tag in plasmids pNG4110, pNG4111, and pNG4210 resulted in plasmids pNG4110A, pNG4111A, and pNG4210A, respectively. For the construction of plasmids pNG4110A and pNG4111A, primers AVI-tagNotI.fw and AVI-tagNotI.rev were annealed to obtain a double-stranded DNA fragment with *Not*I-compatible sticky ends, which was ligated to the *Not*I-linearized plasmids pNG4110 and pNG4111. In the same manner, primers AVI-tagBamHI.fw and AVI-tagBamHI.rev were annealed and ligated to the *Bam*HI-linearized plasmid pNG4210, resulting in plasmid pNG4210A. To construct *lytM* and *sle1*-expressing plasmids, PCR products amplified from *S. aureus* USA300 genomic DNA with primers indicated in Table 2 were digested with *Bam*HI and *Not*I and ligated to *Bam*HI/*Not*I-linearized vectors. Primer combinations using a reverse primer with a stop codon to prevent read-through translation into the plasmid sequences (F1/R2) were used to amplify fragments for ligation to plasmids pNG4110, pNG4111, pNG4110S, and pNG4111S, and primer combinations without a stop codon in the reverse primer (F1/R1) were used to amplify fragments for ligation into plasmids pNG4210, pNG4210S, pNG4110A, pNG4111A, and pNG4210A.

### Protein expression, purification, and detection

For protein expression, lactococcal cultures were induced in the exponential phase of growth at an optical density at 600 nm (OD_600_) of 0.5 by the addition of nisin (final concentration 3 ng/ml; Sigma-Aldrich, St. Luis, MO) and harvested after overnight incubation. Cells were then separated from the growth medium by centrifugation. To concentrate protein samples, nisin-induced culture supernatants were precipitated with 10% TCA. The precipitated proteins were resuspended in lithium dodecyl sulfate (LDS) gel-loading buffer (Life Technologies, Grand Island, NY, USA) to denature them prior to LDS-polyacrylamide gel electrophoresis (PAGE). The respective cells were disrupted with 0.1 μm glass beads in LDS sample buffer (Biospec Products, Bartlesville, USA) in a Precellys 24 homogenizer (Bertin Technologies, Saint Quentin en Yvelines Cedex, France). To visualize the extent to which proteins were secreted, secreted and cellular proteins were analyzed by LDS-PAGE using NuPAGE gels (Life Technologies). Proteins were either visualized using Simply Blue Safe Staining (Life Technologies) or by Western blotting on Protan nitrocellulose membranes (Whatman, Germany) using mouse anti-His_6_-tag (Life Technologies), anti-Strep tag II (Iba Lifesciences, Germany), or anti-AVI-tag (Genscript, Piscataway, USA) primary antibodies. Fluorescent secondary antibodies (goat anti-mouse IRDye 800 CW, LI-COR Biosciences, Lincoln, NE, USA) were used for visualization of bound primary antibodies with an Odyssey Infrared Imaging System (LI-COR Biosciences). Expressed Strep-tag II proteins were purified from growth medium fractions (adjusted to pH 8 with NaOH) using a Strep-Tactin Sepharose 50% suspension following the manufacturer’s protocol (Iba Lifesciences). AVI-tagged Sle1 proteins in cell fractions (Sle1-AVI pellets) were washed twice with phosphate-buffered saline (PBS) and labeled directly with biotin using the BirA biotin ligase (Avidity, Aurora, CO, USA). Cell pellets were then washed twice with PBS and incubated with 0.1 mg Cy3-streptavidin (GE Healthcare Europe, Germany) in PBS for 30 min. After two washes with PBS, pellets were incubated in 6 M urea for 10 min and cell-bound Cy3-Sle1 released to the supernatant was collected after centrifugation. To observe possible non-specific binding of Cy3-streptavidin to biotinylated native *L. lactis* proteins, Sle1-AVI pellets were washed twice with PBS and incubated with 0.1 mg Cy3-streptavidin in 6 M urea for 10 min. After centrifugation, the supernatant was collected and used as Cy3 negative control supernatant.

### Enzyme-linked immunosorbent assays

Strep-tactin-coated microtiter plates (8-well strips, Iba Lifesciences) were incubated for 1 h at room temperature with nisin-induced filter-sterilized growth medium samples (100 μl/well, adjusted to pH 8 with NaOH) containing LytM Strep-tag II fusion proteins. Serial dilutions of previously collected human plasma samples (500–2000,000) were made in PBS, 0.05% Tween 20, 5% skim milk. The plasma samples used were obtained from a patient with epidermolysis bullosa (EB01) and a healthy control volunteer (Control 02) as previously described (van der Kooi-Pol et al. 2013). Specific anti-human IgG secondary antibodies coupled to horseradish peroxidase (dilution 1:2000, Southern Biotechnology, Birmingham, AL) were used according to the manufacturer’s recommendations. Horseradish peroxidase activity was quantified by measuring the hydrolysis of the substrate (O-Phenylenediamine, Sigma-Aldrich) at an optical density of 492 nm (OD_492_) in a plate reader (Biotek Powerwave XS2, USA). Titers were expressed in arbitrary units (AU) obtained by calculating in Excel the extrapolated initial absorbance at serum dilution 1:1 (titer), using a linear regression equation adjusted through the data points of dilutions with measured OD_495_ readings between 1.0 and 0.1 (*SLOPE*(*sample OD readings data set*, *serum dilution factor data set*) *+ INTERCEPT*(*sample OD readings data set*, *serum dilution factor data set*)), with all *R* ^2^ (Pearson product moment correlation coefficient) values > 0.98. Estimated titers of duplicates were averaged.

### Microscopy

*S. aureus* NCTC8325 was grown overnight in TSB and used to inoculate fresh medium (1:50) in the morning. This culture was then grown until the mid-exponential phase. When an OD_600_ of 1.0 was reached, 1 ml of bacterial culture was collected, washed twice with PBS, and incubated in 1.5 ml of PBS containing 1.5 μg (30 μl) of Cy3-Sle1 or Cy3 control supernatant for 1 h at room temperature. After washing three times with PBS, cells were spotted onto polylysine-coated glass slides. Microscopic images were recorded using a Leica DM5500B epifluorescence microscope equipped with Cy3 filter block and a Leica DFC365FX camera using a ×63 objective (Leica Microsystems BV, The Netherlands). The presence of Sle1 was assayed using rabbit anti-Sle1 antibodies (originally referred to as anti-Aaa antibodies) at a 1:500 dilution using an earlier described protocol (Campo et al. 2004). The anti-Sle1/Aaa antibodies were kindly provided by Christine Heilmann (Heilmann et al. 2005). Secondary Oregon Green anti-rabbit antibodies (Molecular Probes) were used at a dilution of 1:850.

### Ethics statements

Plasma from an epidermolysis bullosa patient and a healthy volunteer was collected under the approval of the medical ethics committee of the University Medical Center Groningen (approval no. NL27471,042,09) upon written informed consent and with adherence to the Helsinki Guidelines (van der Kooi-Pol et al. 2013). The necessary written informed consent was obtained from both plasma donors.

## Results

### Development of vectors for the secretion of Strep- or AVI-tagged proteins from *L. lactis*

In order to express and secrete AVI-tag or Strep-tag fusion proteins from *L. lactis*, the vector set pNG4110/111/210 was modified as depicted in Fig. 1. In a previous study, these vectors were successfully used for the extra-cytoplasmic production and purification of heterologous His_6_-tagged proteins (Neef et al. 2015). The His_6_-tag-encoding sequences in the vectors pNG4110/111/210 were replaced by the Strep-tag-encoding sequence, generating the vectors pNG4110S/111S/210S (Fig. 1c). Respectively, these vectors can be used to express proteins with N-terminal, N-terminal and TEV-cleavable, or C-terminal Strep-tags. Further, the AVI-tag-encoding sequence was added in the vectors pNG4110/111/210, respectively, generating vectors pNG4110A/111A/210A, which contain both the His_6_-tag- and AVI-tag-encoding sequences (Fig. 1d). Sequencing of the different vectors with inserted Strep- or AVI- tags showed that all had the expected nucleotide sequences and, upon culturing over multiple generations, no deletions were observed (data not shown). With respect to usage of the AVI-tag, it is noteworthy that BLAST analysis with the 15-residue AVI-tag amino acid sequence showed that none of the genes of *L. lactis* NZ9000 (i.e., the parental strain of PA1001) encodes proteins with similarity to the AVI-tag. This renders false-positive biotinylation of *L. lactis* proteins by the BirA enzyme unlikely. Lastly, it should be noted that the restriction sites used for cloning will add additional residues to an expressed protein of interest. This may have consequences for particular applications of the expressed protein.

### Production of Strep- or AVI-tagged fusions of the staphylococcal proteins LytM and Sle1

**Fig. 2:**
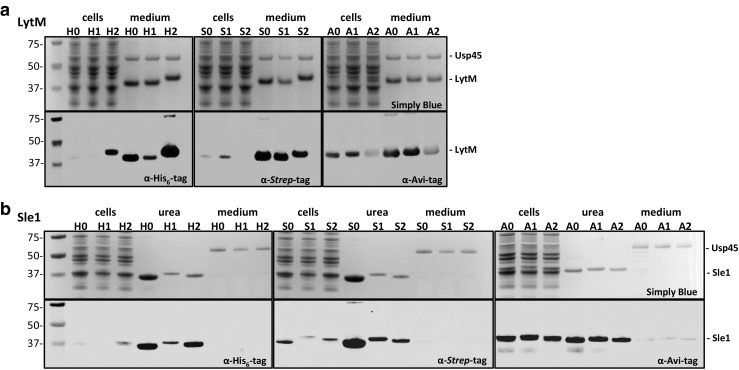
Expression of various tagged derivatives of the *S. aureus* LytM and Sle1 proteins. Detection of LytM (**a**) and Sle1 (**b**) expression in *L. lactis* by LDS-PAGE and subsequent Simply Blue staining (upper panels) or Western blotting (lower panels) using antibodies against different tags (α-His_6_-tag, α*-*Strep-tag, α-Avi-tag). Expression of the AVI-tagged fusion proteins was also verified using α-His_6_-tag antibodies showing a similar pattern of expression (results not shown). Expression from different vectors is indicated as follows: H0, pNG4110; H1, pNG4111; H2, pNG4210; S0, pNG4110S; S1, pNG4111S; S2, pNG4210S; A0, pNG4110A; A1, pNG4111A; and A2, pNG4210A. Lanes loaded with cell or growth medium fractions are indicated. Urea, supernatant fraction obtained after incubation of cells with 6 M urea and centrifugation. It should be noted that, in case of Sle1 production (**b**), the cell fractions correspond to cell fractions after urea incubation. Molecular weights of marker proteins are indicated on the left and the positions of LytM and Sle1 fusion proteins or the major secreted protein of *L. lactis* (Usp45) are indicated on the right

To test our newly constructed “third-generation” vector set for protein production in *L. lactis*, genes encoding the naturally exported *S. aureus* proteins LytM and Sle1 were cloned into all of these vectors. The resulting plasmids were then introduced and expressed in *L. lactis* PA1001. The PA1001 strain lacks the gene for the major peptidoglycan hydrolase AcmA due to which cells do not lyse or separate during and after growth (Steen et al. 2005). This lack of separation results in a sedimentation of cells during growth in a standing culture. To obtain maximal protein production, 2% glucose with aeration was used to obtain a higher cell density compared to 0.5% glucose without aeration. Further, the PA1001 strain displays reduced proteolytic activity due to deletion of the *htrA* gene (Neef et al. 2014). After induced expression, all fusion proteins were produced as demonstrated by LDS-PAGE, where the respective proteins showed a mobility that matched their expected sizes (Fig. 2).

LytM is a peptidoglycan hydrolase with glycyl-glycine endopeptidase activity that is secreted by all *S. aureus* strains. In previous studies, LytM was shown to be a very immunogenic protein of *S. aureus.* This was determined using different techniques, such as enzyme-linked immunosorbent assays (ELISA) (van den Berg et al. 2015) and Luminex bead-based flow cytometry (van der Kooi-Pol et al. 2013). The presently constructed LytM fusion proteins were observed mainly in the growth medium fractions (Fig. 2a). While expression and secretion of the three differently His_6_-tagged LytM proteins were comparable as shown by Simply Blue staining, immunodetection of LytM with the C-terminal His_6_-tag was most efficient and lowest for LytM with an N-terminal His_6_-tag and TEV site. An opposite effect was observed for immunodetection of the AVI-tagged variants as expressed from the plasmids pNG4210A and pNG4111A (Fig. 2a). Furthermore, depending on the tag and its position, different amounts of LytM remained attached to the cells. This suggests that the nature of the tag and its N- or C-terminal position influence the expression, secretion, and/or detection efficiency of the fusion products.

Sle1 is a 32-kDa N-acetylmuramyl-l-alanine amidase which is involved in cell separation (Kajimura et al. 2005). The protein consists of a C-terminal CHAP domain (PF05257), which is responsible for peptidoglycan hydrolysis, plus three N-terminal LysM domains (PF01476) that are responsible for non-covalent cell wall-binding (Dreisbach et al. 2011; Visweswaran et al. 2014). Localized cell surface-binding of this protein has been shown using fusions to the fluorescent mCherry protein (Frankel and Schneewind 2012). Accordingly, all Sle1 fusion proteins were detected in the cell fractions upon nisin-induced expression. Release of Sle1 from the collected cells was achieved by incubation with 6 M urea, which disrupts the interaction of the Lys domains with the cell wall and results in the subsequent release of Sle1 (Fig. 2b). The urea-released Sle1 was effectively recovered by centrifugation. Of note, upon LDS-PAGE and Simply Blue staining, no detectable amounts of proteins other than Sle1 were observed in the respective supernatant fraction. As was observed for the LytM fusion proteins, the expression levels of Sle1 fusion proteins varied depending on the nature and position of the tag. In the case of Sle1 with a C-terminal AVI-tag, hybridizing bands with apparently lower molecular weight were detected upon expression from vectors pNG4110A and pNG4111A (Fig. 2b). Most likely, these relate to degradation products of the respective Sle1 fusions. In all other cases, Sle1 was apparently stably produced. These data show that all fusion products could be produced form the newly constructed vector sets.

### Application of Strep-tagged LytM in an ELISA

**Fig. 3:**
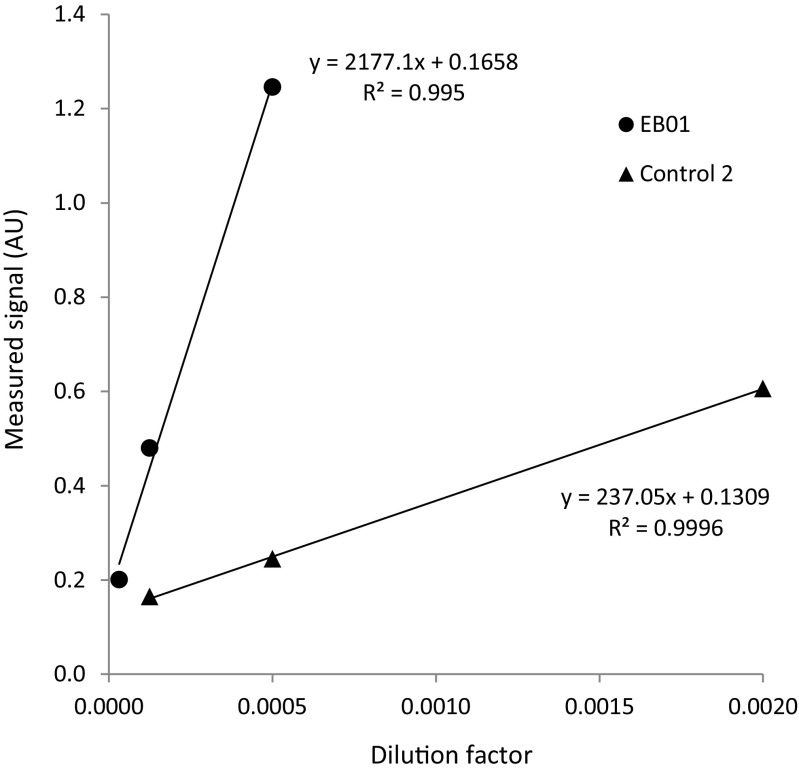
ELISA of Strep-tagged LytM using human plasma. Strep-tagged LytM was bound to a Strep-tactin 96-well microtiter plate by applying growth medium fractions of *L. lactis* pNG4110S-*lytM*. Upon washing of the plate, ELISA was performed using the human plasma samples EB01 and Control 2 as indicated. Regression equations, trend lines (black), and *R* ^2^ errors are indicated

To test the application potential of secreted Strep-tagged staphylococcal proteins for the detection of specific human antibodies, we applied an ELISA approach. To this end, the Strep-tagged LytM fusion protein was bound to Strep-tactin-coated microwell plates. The Strep-tagged LytM expressed from plasmid pNG4110S-*lytM* was selected for this application because this Strep-tagged LytM was slightly better detectable than the Strep-tagged LytM expressed from plasmids pNG4111S-*lytM* and pNG4210S-*lytM* (Fig. 2a). Specifically, nisin-induced culture supernatants of pNG4110S-*lytM* were added to microtiter plate wells coated with Strep-tactin. The plates were subsequently washed and ELISA was performed using plasma from an epidermolysis bullosa patient (EB01) and an age-matched healthy control individual (Control 2), both of which had been previously described (van der Kooi-Pol et al. 2013). The results as presented in Fig. 3 show that the EB01 plasma contained a substantially higher level of anti-LytM IgG (2177.5 AU) than the Control 2 plasma (237 AU), which is in full agreement with our previous observations. This shows that Strep-tagged fusions of *S. aureus* proteins expressed and secreted in *L. lactis* can be directly recovered from growth medium fractions and used for ELISA.

### Use of the AVI-tag to assess localized Sle1-binding to *S. aureus* cells

**Fig. 4:**
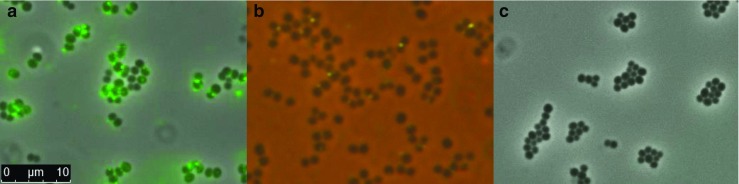
Binding of AVI-tagged Cy3-labeled Sle1 to *S. aureus* NCTC8325 cells. **a** Fluorescence microscopy of *S. aureus* NCTC8325 cells upon incubation with AVI-tagged Cy3-labeled Sle1. **b** *S. aureus spa sbi* double-mutant cells incubated with Sle1-specific rabbit antibodies and secondary Oregon Green anti-rabbit antibodies. **c** Negative control of cells incubated with non-biotinylated and therefore not fluorescently labeled AVI-tagged Sle1

A Sle1 fusion protein with an N-terminal His_6_-tag and a C-terminal AVI-tag was obtained upon expression from pNG4110A-*sle1*. Of note, this fusion protein fractionated with the expressing cells, which is in accordance with the presence of three cell wall-binding LysM domains in this protein (Fig. 2b). To assess whether the cell-associated AVI-tagged Sle1 can be directly labeled with biotin, the producing *L. lactis* cells were collected and incubated with biotin and the BirA ligase. Subsequently, the cells were washed and incubated with Cy3-streptavidin. The resulting Cy3-Sle1 was released from the *L. lactis* cells by incubation with 6 M urea. Lastly, upon centrifugation, the supernatant fraction containing Cy3-Sle1 was diluted 50-fold and added to *S. aureus* NCTC8325 cells. Fluorescence microscopy showed that Cy3-labeled Sle1 bound locally on the surface of *S. aureus* NCTC8325 cells with a preference for the septal region (Fig. 4a). Similar hotspots for binding were observed when using Sle1-specific antibodies in combination with a secondary Oregon Green-labeled antibody (Fig. 4b). In this case, a *S. aureus* Δ*spa* Δ*sbi* double-mutant strain was used for the immunodetection in order to avoid Fc-specific IgG-binding by the staphylococcal protein A (Spa) and the Sbi protein of *S. aureus*. Of note, a possible interference by protein A and Sbi is not an issue when using the Cy3-labeled AVI-tagged Sle1 fusion protein. To control for possible Cy3-labeling of biotinylated native *L. lactis* proteins, *L. lactis* cells expressing the AVI-tagged Sle1 fusion protein were incubated as described above but in the absence of the BirA ligase. Upon extraction of the AVI-tagged Sle1 from the *L. lactis* cells and subsequent incubation with *S. aureus* NCTC8325 cells, no labeling of the staphylococcal cells was observed (Fig. 4c). This shows that an AVI-tagged protein produced in *L. lactis* can be effectively labeled with biotin and Cy3-streptavidin for further applications.

## Discussion

In this study, we describe a third-generation set of cloning vectors for the expression and secretion of differently tagged heterologous proteins from *L. lactis*. These vectors enable isolation of proteins under mild conditions using Strep-tag fusions or site-specific labeling with biotin using AVI-tag fusions. For both types of tags, N-terminal or C-terminal fusion proteins can be produced as was shown with the reporter proteins LytM and Sle1 from *S. aureus*. A secreted Strep-tag fusion of the staphylococcal protein LytM was successfully used for rapid immune screening using human sera. LytM was selected for this purpose because it was previously shown to be highly immunogenic (van der Kooi-Pol et al. 2013; van den Berg et al. 2015). An AVI-tagged variant of the staphylococcal Sle1 protein was site-specifically labeled and used for detection of localized binding on staphylococcal cells because it was previously shown to display a particular localization pattern on the *S. aureus* cell surface (Frankel and Schneewind 2012).

The combination of nisin-inducible expression using the *L. lactis* PA1001 strain allowed for controlled stable overnight expression of all fusion proteins. As expected based on the autolysin- and protease-deficiency of the PA1001 strain, no autolysis was detectable for any of the fusion protein-expressing derivative strains, and product degradation appears to be negligible. Only a minor possible degradation product of AVI-tagged Sle1 proteins was detectable upon overnight expression. This is in agreement with earlier reports on the usage of this strain, where a possible involvement of cytoplasmic or intramembrane proteases was invoked in residual degradation of the protein ClfB (Neef et al. 2015). These results led to the conclusion that *L. lactis* might have an additional proteolytric acitvity wich has a substrate specificity and thus does not degrade all secreted proteins. Possibly, Sle1 is like ClB, one of the substrates for this yet unknown protease. Because of this variation in expression, secretion, and stability is protein dependent, we generated a vector set for possible fusion of tags at either end of a protein to determine after detection of expression which fusion can be used best for its purpose.

Measurements of human immune responses against *S. aureus* LytM were previously performed by ELISA (van den Berg et al. 2015) and Luminex bead-based flow cytometry (van der Kooi-Pol et al. 2013). In both cases, the analyses involved the purification of His_6_-tagged LytM and subsequent binding of the purified protein to ELISA plates or Luminex beads. As shown in our present study, expression of LytM with a Strep-tag obviates the purification step as Strep-tagged LytM secreted by *L. lactis* can be directly applied to Strep-tactin-coated microplate wells for LytM immobilization on the plates. Importantly, the recorded human IgG-binding by immobilized LytM matched well with the previously published data, where it was shown that plasma samples from *S. aureus*-colonized epidermolysis bullosa patients contained significantly higher levels of anti-LytM IgGs than plasma samples from healthy control individuals (van der Kooi-Pol et al. 2013; Swierstra et al. 2015).

Previously, the microscopic detection of non-covalently cell wall-bound *S. aureus* proteins has been performed in various different ways, including immunofluorescence with labeled antibodies (Steen et al. 2003), *in-frame* fusions to fluorescent proteins like mCherry (Frankel and Schneewind 2012) or GFP (Visweswaran et al. 2013), or re-binding of purified proteins that had been randomly labeled with fluorophores (Zoll et al. 2012). Of note, such approaches may have certain drawbacks. In particular, in immunofluorescence microscopy, the presence of the IgG-binding proteins Spa (protein A) and Sbi results in an Fc-dependent off-target signal. This can be overcome by using *spa sbi* mutant strains but it precludes analyses with clinical *S. aureus* isolates. The use of *in-frame* fusions between a protein of interest and fluorescent proteins may be hampered by low signal intensities due to low expression levels, possible misfolding of the fluorescent protein, or incompatibilities with the protein secretion machinery (Chudakov et al. 2010). Further, random labeling of proteins used in re-binding studies can potentially interfere with the binding function of the protein. Our present results show that the AVI-tag allows for direct labeling of AVI-tagged Sle1 protein with Cy3-streptavidin. The resulting fluorescently marked Sle1 protein was then used for re-binding studies with staphylococcal cells. Due to the presence of the three LysM peptidoglycan-binding domains, the Sle1 protein binds to the cell wall upon interaction. Previously, Frankel and Schneewind 2012 used mCherry fused to Sle1 for *S. aureus* cell wall-binding studies to demonstrate the septal binding of Sle1. In our present approach, the previously observed preferential septal binding of Sle1 was clearly reproduced, indicating that the AVI-tag can be used as an alternative to in-frame fusions with fluorescent proteins. Of note, with the AVI-tagged Sle1, we also obtained possible evidence for additional loci where Sle1 may bind to the *S. aureus* cell surface besides the septal region (Fig. 4a). The latter observation deserves further investigations to identify the specific nature of the respective interactions and their possible biological relevance.

In conclusion, we have developed a set of third-generation expression vectors that enhances the versatility of *L. lactis* as a system for the production of proteins carrying tags that can be used for affinity purification and site-specific protein labeling.
